# Structural Basis of Latrophilin-FLRT Interaction

**DOI:** 10.1016/j.str.2015.01.013

**Published:** 2015-04-07

**Authors:** Verity A. Jackson, Daniel del Toro, Maria Carrasquero, Pietro Roversi, Karl Harlos, Rüdiger Klein, Elena Seiradake

**Affiliations:** 1 Department of Biochemistry, Oxford University, South Parks Road, Oxford OX1 3QU, UK; 2 Max-Planck Institute of Neurobiology, Am Klopferspitz 18, 82152 Munich-Martinsried, Germany; 3 Division of Structural Biology, Oxford University, Roosevelt Drive, Oxford OX3 7BN, UK; 4 Munich Cluster for Systems Neurology (SyNergy), Feodor-Lynen-Straße 17, 81377 Munich, Germany

## Abstract

Latrophilins, receptors for spider venom α-latrotoxin, are adhesion type G-protein-coupled receptors with emerging functions in synapse development. The N-terminal region binds the endogenous cell adhesion molecule FLRT, a major regulator of cortical and synapse development. We present crystallographic data for the mouse Latrophilin3 lectin and olfactomedin-like (Olf) domains, thereby revealing the Olf β-propeller fold and conserved calcium-binding site. We locate the FLRT-Latrophilin binding surfaces by a combination of sequence conservation analysis, point mutagenesis, and surface plasmon resonance experiments. In stripe assays, we show that wild-type Latrophilin3 and its high-affinity interactor FLRT2, but not the binding-impaired mutants we generated, promote HeLa cell adhesion. In contrast, cortical neurons expressing endogenous FLRTs are repelled by wild-type Latrophilin3 and not by the binding-impaired mutant. Taken together, we present molecular level insights into Latrophilin structure, its FLRT-binding mechanism, and a role for Latrophilin and FLRT that goes beyond a simply adhesive interaction.

## Highlights

• The LPHN olfactomedin-like domain forms a five-bladed β propeller
• A conserved calcium-binding site is located at the center of the protein
• Latrophilin-FLRT binding depends on a conserved binding site
• Mutations in the binding site inhibit Latrophilin-FLRT signaling

## Graphical Abstract

**Figure:**
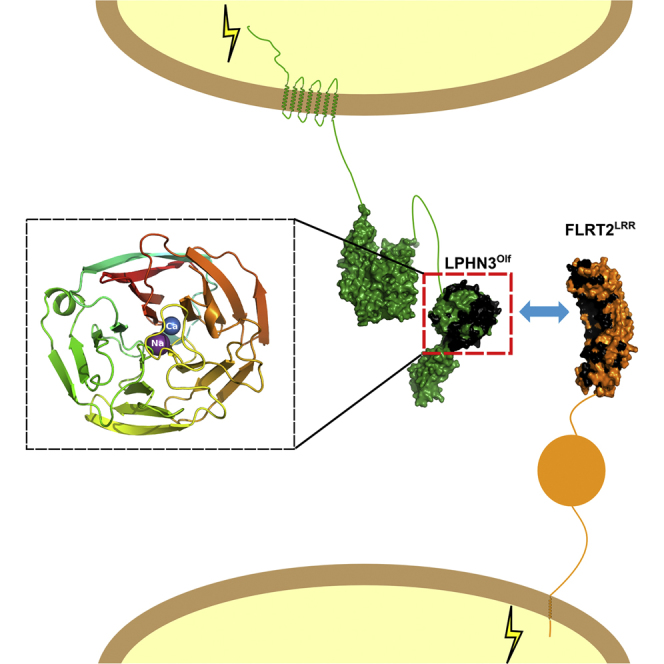

## Teaser

Jackson et al. describe a crystal structure of mLPHN3 lectin and olfactomedin-like (Olf) domains, revealing the Olf β-propeller fold and calcium-binding site. Assays using HeLa cells and cortical neurons reveal a bi-functional role for Olf and its ligand FLRT, leading to HeLa cell adhesion and neuron repulsion.

## Introduction

Latrophilins (LPHN1–3) are a family of adhesion type G-protein-coupled receptors (GPCRs) first characterized as calcium-independent receptors for the black widow spider toxin α-latrotoxin, which causes massive exocytosis of calcium at nerve terminals (Davletov et al., 1996; Krasnoperov et al., 1996; Lelianova et al., 1997; Sugita et al., 1998). More recently, LPHNs were shown to function as heterophilic cell adhesion molecules in processes such as synapse formation or maintenance. All LPHNs have a large ectodomain of approximately 1,000 amino acids, with an N-terminal lectin domain, followed by an olfactomedin-like (Olf) domain, a serine/threonine-rich region, HormR domain, and a GPCR-autoproteolysis inducing (GAIN) domain containing a GPCR proteolysis site (GPS). The structure of the N-terminal lectin domain was solved a few years ago using nuclear magnetic resonance (NMR) (Vakonakis et al., 2008). It folds into a β sandwich comprising two antiparallel sheets decorated with a 10-residue long α helix and two extended loops. One of the loops harbors a low-affinity rhamnose-binding site (Vakonakis et al., 2008). More recently, the crystal structure of the HormR and GAIN domains was solved by X-ray crystallography (Araç et al., 2012). The structure revealed the basis for the autoproteolytic cleavage of LPHNs at the GPS via an evolutionarily ancient mechanism (Araç et al., 2012). Within the LPHN ectodomain, the Olf domain is the only predicted globular domain that remains structurally uncharacterized.

LPHN1 and 3 are expressed predominantly in the brain. In humans, mutations in LPHN3 are associated with the largely hereditary attention-deficit hyperactivity disorder (ADHD) (Arcos-Burgos et al., 2010; Domené et al., 2011). In zebrafish, decreased activity of the LPHN3 homolog elicits ADHD-like behavior (Lange et al., 2012). The endogenous functions of LPHNs are linked to cell adhesion and synapse formation or maintenance, but the molecular mechanisms underlying these functions are only beginning to emerge. Known adhesive ligands of LPHNs include members of the teneurin family (Silva et al., 2011), neurexins (Boucard et al., 2012), and the fibronectin leucine-rich repeat transmembrane proteins (FLRT1–3) (O’Sullivan et al., 2012). FLRTs have recently emerged as powerful guidance factors in vascular, neural, and early embryonic development (Egea et al., 2008; Leyva-Díaz et al., 2014; Maretto et al., 2008; Müller et al., 2011; O’Sullivan et al., 2012; Seiradake et al., 2014; Yamagishi et al., 2011). They promote both cell adhesion (through homophilic interactions) and cell repulsion (through interaction with Uncoordinated-5 [Unc5] receptors). Due to their dual functionality, FLRTs are also referred to as repulsive cell adhesion molecules (ReCAMs) (Seiradake et al., 2014). LPHN3 and FLRT3 were reported to interact in *trans* through their ectodomains to mediate cell adhesion, an interaction that promotes the development of glutamatergic synapses (O’Sullivan et al., 2012). The LPHN Olf domain is required for the synapse-promoting function and also for FLRT binding (O’Sullivan et al., 2014).

Olf domains are present in at least 13 different proteins in mammals including the LPHNs (1–3), noelins (1–3; also called olfactomedins 1–3), olfactomedin 4, olfactomedin-like (1–3; with olfactomedin-like 2 separated into 2A and 2B), myocilins, and gliomedins (Tomarev and Nakaya, 2009); however, the lack of structural information has hampered their functional analysis. Here we present the crystal structure of an N-terminal fragment of murine LPHN3 comprising the lectin and Olf domains (hereafter LPHN3^Lec-Olf^). We find that the Olf domain folds into a five-bladed β propeller with metal ions buried in the center of the molecule. We show that, by introducing specific mutations at the surface of the Olf domain, we can reduce or impair binding to FLRT. We also show that LPHN3^Lec-Olf^ is sufficient to attract FLRT-expressing cells through its adhesive FLRT-binding properties. Interestingly, the FLRT-binding site on LPHN3 is required for repulsive activity of LPHN3 toward cortex-derived neurons. The structural data presented here suggest that LPHN3 is a bi-functional protein with adhesive and repulsive properties, possibly mediated by similar surfaces of the Olf domain.

## Results

### Crystal Structure of the Murine LPHN3^Lec-Olf^

**Figure 1:**
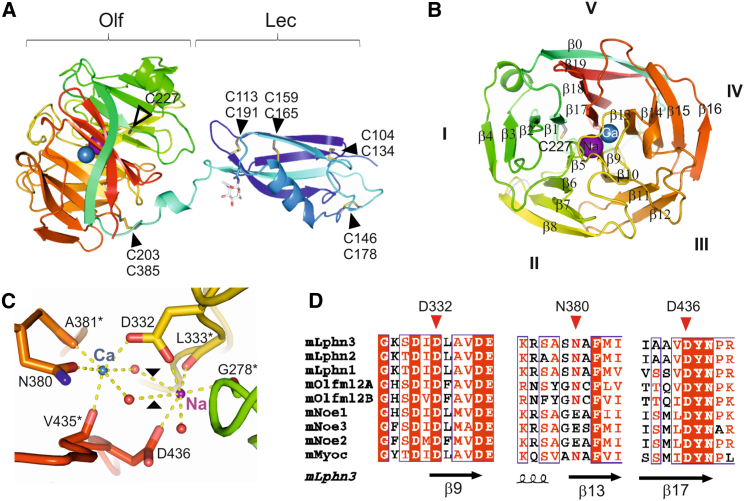
Crystal Structure of mLPHN3^Lec-Olf^ (A) The structure is shown as cartoon model colored according to the rainbow (blue = N terminus, red = C terminus). Disulfide bridges are labeled, shown as yellow sticks and marked with black arrowheads. The unpaired cysteine C227 located at the center of the Olf domain is marked with a white arrowhead. Predicted sodium and calcium ions are shown as purple and blue spheres, respectively. (B) The structure is shown as in (A), but rotated by ∼90° presenting a view of the Olf domain along the pseudo-5-fold symmetry axis. Individual β sheets are numbered (I–V) and β strands are labeled (β0–19). (C) Zoomed view of the calcium (Ca, blue) and sodium (Na, purple) binding sites in the Olf domain. Coordinating water molecules are shown as red spheres. Coordinating residues are shown as sticks. Asterisks mark residues for which only selected backbone atoms are shown as sticks. Dotted lines show polar contacts for the calcium and sodium ions, as predicted by PyMol. Two of the indicated contacts are above 3.3 Å in distance and were therefore not included in the CBVS calculation. These two contacts are marked with black arrowheads. (D) The sequence alignment of selected Olf domain containing proteins reveals that mLPHN3 D332 and D436 are conserved among other mLPHNs, mouse noelins (mNoe1–3), mouse olfactomedin-like 2A and 2B (mOlfm2A, 2B) and mouse myocilin (mMyoc). N380 is replaced by a glutamic acid in noelins. See also Figure S1.

We expressed secreted murine LPHN3^Lec-Olf^ in human embryonic kidney (HEK) 293T cells using established protocols to circumvent the formation of heterogeneous glycans (Aricescu et al., 2006), and determined the crystal structure to a resolution of 2.16 Å (Table S1). The structure reveals two globular domains separated by a 17-residue linker (residues V192–K208) (Figure 1A). The structure of the LPHN3 lectin domain agrees closely with the lectin domain of LPHN1 solved using solution NMR (Vakonakis et al., 2008) (root-mean-square deviation [rmsd] is 0.86 Å for 90 aligned C_α_ atoms). The Olf domain folds into a five-bladed β propeller, with each blade consisting of a four-stranded β sheet (Figure 1B). The five blades (I–V) are arranged successively in an anticlockwise direction around the central channel, giving rise to the disk-like architecture of the propeller. The β strands within each blade exhibit an up-and-down connectivity. The first strand packs against the first strand of the other blades in a parallel orientation to form a central channel. The fourth strands from blades I–IV largely form the periphery of the disk together with the Olf domain N terminus (β0), which contributes as the fourth strand to blade V. A disulfide bridge (C203–C385) connects the region upstream of β0 with blade IV and appears to stabilize β0 in its position. Notably for an extracellular protein, LPHN3^Lec-Olf^ contains a reduced cysteine (C227) buried in the core of the Olf domain. This cysteine is conserved in the three murine LPHNs, but not in other Olf domains (Figure S1A). The loops between the β strands within a sheet are generally short on the lectin-exposed side, with the exception of the β3–β4 loop which folds into an 8-residue helix sandwiched by blades I and V. The loops on the other side of the propeller are of varying length, the longest of which connects β8 and β9 and occludes the entrance to the central ion-containing channel.

In the electron density map, two potential metal atoms were visible approximately halfway through the central Olf channel. We analyzed the coordination patterns of these ions and calculated calcium bond-valence values (CBVS) to infer their chemical identities (Müller et al., 2003). The result of this analysis points to calcium (CBVS = 1.767) and sodium (CBVS = 1.560) for the octahedrally coordinated and pentacoordinated ion, respectively. The calcium ion equatorial ligands are two water molecules, the backbone carbonyl oxygen of A381, and the oxygen of the carboxamide N380. The backbone carbonyl oxygen of V435 and the carboxylic oxygen of D332 occupy the apical coordination sites (Figure 1C). The sodium ion is coordinated by the backbone carbonyl oxygens of G278 and L333 in addition to the carboxylic oxygens of D332 and D436, with the remaining coordination site filled by a water molecule (Figure 1C). Two additional water molecules are further away, shared with the calcium ion, and are not strictly speaking first-sphere coordination ligands of the sodium ion. The three amino acid side chains involved in coordinating the metal ions, D332, N380, and D436, are highly conserved (Figure 1D) among LPHNs, myocilin, olfactomedin-like 2A/B, and, to some extent, noelins, suggesting that the Olf domains from these proteins could also contain metal-binding sites.

Comparison of the LPHN3^Lec-Olf^ structure with structures in the Dali database (Holm and Rosenström, 2010) reveals highest structural homology with a range of enzymes, especially the type I glutaminyl cyclases (QCs) from the plant pathogenic bacterium, *Xanthomonas campestris* (PDB accession code 3MBR) (Huang et al., 2010); the rmsd is 3.2 Å for 205 aligned C_α_ atoms. The catalytic site of this enzyme also contains a calcium ion in the central channel of the propeller (Figures S1B and S1C).

### Mapping and Mutagenesis of the LPHN-FLRT-Binding Site

**Figure 2:**
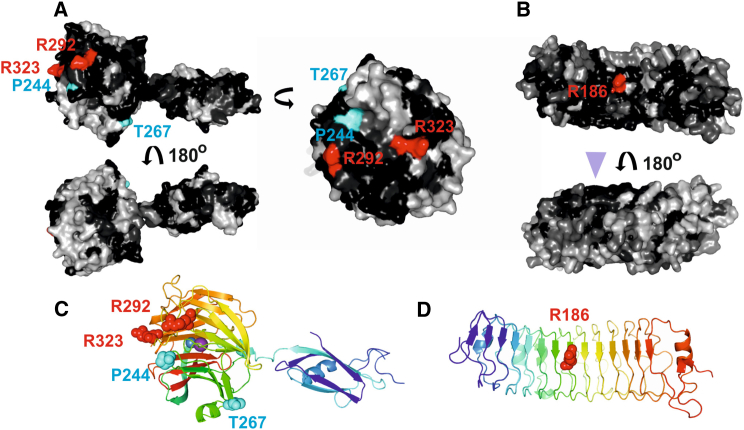
Surface Sequence Conservation Analysis and Mutagenesis (A) The structure of LPHN3^Lec-Olf^ is shown as surface views. A sequence alignment of mouse LPHN3 and 1, chicken LPHN2, and LPHN from fish (*Danio rerio*) and frog (*Xenopus tropicalis*) was used to produce surface conservation scores for LPHN3^Lec-Olf^ with Consurf (Ashkenazy et al., 2010). Black indicates highest conservation scores and white the lowest conservation scores. Residue positions that were mutated to asparagine to introduce an artificial N-linked glycosylation site are labeled and colored red or cyan. (B) The structure of the FLRT2 LRR domain (FLRT2^LRR^) (Seiradake et al., 2014) is shown as surface views. A sequence alignment of FLRT2 and FLRT3 from mouse, frog, and fish was used to produce surface conservation scores. The conserved Unc5-binding site on FLRT2 is marked with a purple arrowhead. Mutants carrying an artificial N-linked glycosylation site at R186 (red) were previously shown to reduce adhesive properties of FLRTs (Seiradake et al., 2014). (C and D) Cartoon views of LPHN3^Lec-Olf^ and FLRT2^LRR^. Colors are according to the rainbow, as in Figure 1. Mutated residues are colored separately and depicted as spheres.

Given the importance of the FLRT-LPHN interaction in brain wiring and synaptic development (O’Sullivan et al., 2012, 2014), and the fact that FLRT and LPHN interactions were observed among multiple homologs (O’Sullivan et al., 2012), we hypothesized that the binding surfaces on FLRT and LPHN would be conserved across species. We generated sequence conservation scores (Glaser et al., 2003) using alignments of FLRTs and Latrophilins from mouse, chicken, frog, and fish, and mapped these onto the structures of LPHN3^Lec-Olf^ and the previously solved FLRT2 leucine-rich repeat (LRR) domain (Seiradake et al., 2014) (Figures 2A and 2B). LPHN3^Lec-Olf^ exhibits a sequence-conserved patch on the Olf domain, stretching across blades II and III of the propeller. Surfaces of blades I, IV, and V are relatively less conserved (Figure 2A). On FLRT2, a conserved surface patch extends from the concave to one lateral side of the LRR domain (Figure 2B). We previously showed that the conserved lateral side harbors the Unc5-binding side while the conserved concave side promotes FLRT-FLRT interaction (Seiradake et al., 2014). To probe these conserved surface regions, we generated a series of mutant proteins in which N-linked glycosylation sites were introduced at various positions. Introduction of N-linked glycans in protein-protein binding sites is an established way of disrupting protein binding (Seiradake et al., 2010, 2011, 2013, 2014). For LPHN3 these mutations were R292N+R294T (LPHN3^LF^), R324N+G326T (LPHN3^LF(2)^), T267N+K269T (LPHN3^LF(3)^), and P244N+R246T (LPHN3^LF(4)^) (Figures 2A and 2C). For FLRT2 we used previously described mutants FLRT2^FF^ and FLRT2^UF^ (Seiradake et al., 2014).

**Figure 3:**
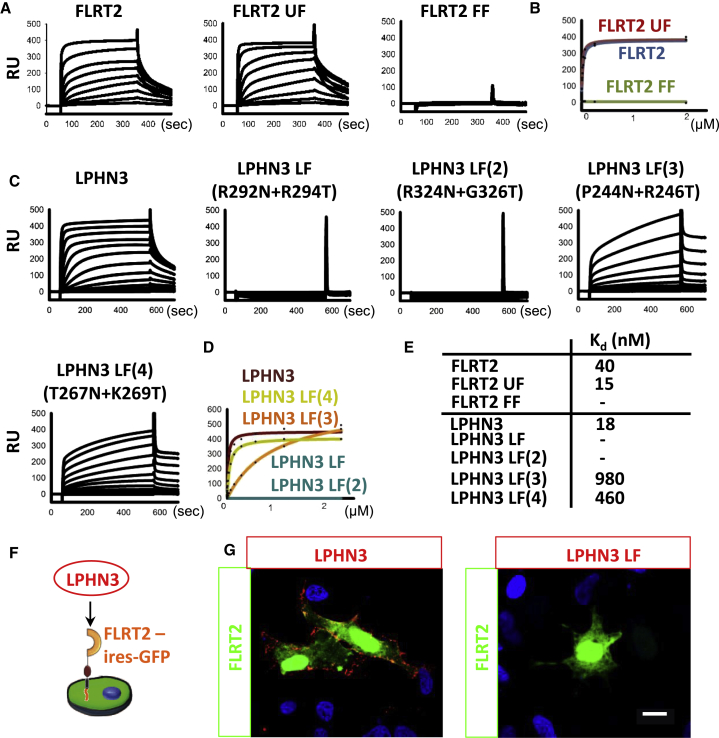
Surface Plasmon Resonance Data Reveal FLRT2-LPHN3 Binding Surfaces (A) Full ectodomains of wild-type murine FLRT2, the non-Unc5-binding mutant FLRT2^UF^ or the non-dimerizing mutant FLRT2^FF^ were immobilized on a CM5 Biacore chip and the purified olfactomedin domain of murine LPHN3 was injected as analyte at different concentrations. Response units are shown for each injection. (B) Binding curves were fitted using maximum response units for each injection and a 1:1 binding model. (C) Biotinylated wild-type or mutant LPHN3^Lec-Olf^ was immobilized on streptavidin-coated Biacore chips and the ectodomain of FLRT2 was injected at different concentrations. Response units are shown as in (A). (D) Binding curves from (C) were fitted using maximum response units for each injection and a 1:1 binding model. (E) Calculated *K*_d_ values are shown for binding of FLRT2 constructs to the LPHN3 olfactomedin domain (top three rows) and for LPHN^Lec-Olf^ constructs binding to FLRT2 ectodomain. (F and G) We tested the binding of wild-type or LF mutant LPHN3^Lec-Olf^ to HeLa cells transfected with a FLRT2-ires-GFP vector (Seiradake et al., 2014). Bound LPHN^Lec-Olf^ proteins were visualized using an antibody against the polyhistidine tag (red). Scale bar, 10 μm.

Surface plasmon resonance (SPR) experiments revealed high-affinity binding between wild-type FLRT2 and LPHN3 proteins, with *K*_d_ values in the range 18–40 nM depending on the experimental setup (Figures 3A–3E). In contrast, the LPHN3^Lec-Olf^ mutants LPHN3^LF^ and LPHN3^LF(2)^ do not have measurable affinity to the FLRT2 ectodomain. LPHN3^LF(3)^ and LPHN3^LF(4)^ bind FLRT2, but with greatly reduced affinity (Figures 3C–3E). We conclude that the FLRT-binding site involves conserved surface regions on blades II and III of the LPHN3 Olf domain. We also tested the binding of previously described FLRT2 mutants to the LPHN3 Olf domain and found that the non-Unc5 binding FLRT mutant (UF) binds with high affinity. In contrast, the non-FLRT-binding FLRT mutant (FF) lost measurable affinity for LPHN3. Thus the same FLRT2 surface that promotes adhesive homophilic interaction also promotes FLRT2-LPHN3 interaction. For subsequent functional analysis we used wild-type and LF mutant LPHN3^Lec-Olf^ proteins. Consistent with the SPR data, wild-type LPHN3^Lec-Olf^, but not the mutant, bound to full-length FLRT2 expressed at the surface of cells (Figures 3F and 3G).

### FLRT2-LPHN3 Interaction Controls Cell Adhesion and Repulsion

**Figure 4:**
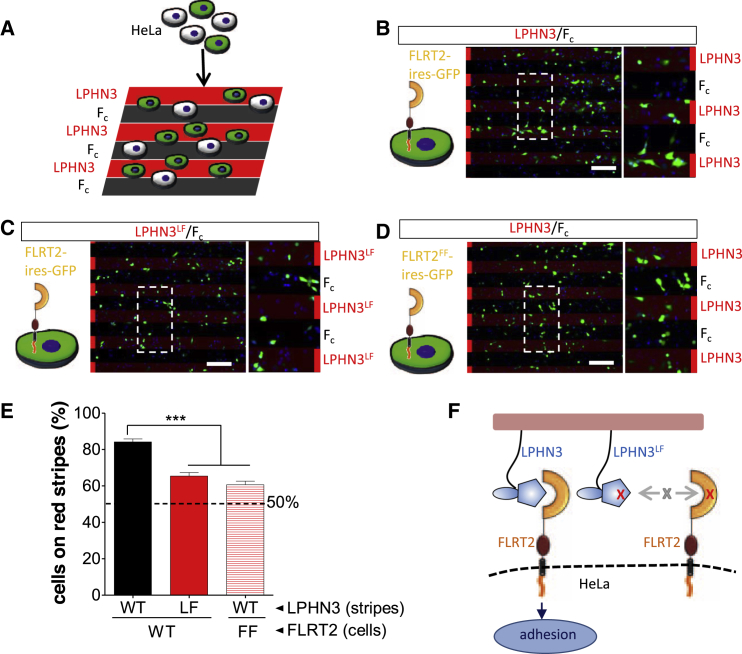
The FLRT-Binding Site on LPHN3^Lec-Olf^ Is Required for HeLa Cell Adhesion (A) Cartoon outlining the experimental setup for HeLa cells grown on alternating LPHN3 and F_c_ stripes. (B–D) Transfected HeLa cells (green) were grown on alternating stripes of wild-type or mutant LPHN3^Lec-Olf^ (red) and F_c_ control proteins (black). Cells expressing FLRT2 are attracted to LPHN3^Lec-Olf^, but not LPHN3^LF^ mutant, stripes. Insets are higher magnification images showing the distribution of cells on the stripes. Red bars indicate the locations of the red (LPHN3-containing) stripes. Cell nuclei were counterstained with DAPI (blue). Scale bar, 200 μm. (E) Quantification of the data shown in (B)–(D). Data were quantified by calculating the percentage of GFP-expressing (FLRT2 or FLRT2^FF^-expressing) cells present on the red (LPHN3- or LPHN3^LF^-containing) stripes. ^∗∗∗^p < 0.001 (wild-type versus LF and FF), one-way ANOVA test with Tukey’s post hoc analysis. Error bars represent the SEM. (F) Cartoon summary depicting how wild-type FLRT2 and LPHN3 proteins, but not the binding-impaired mutants, promote HeLa cell adhesion.

**Figure 5:**
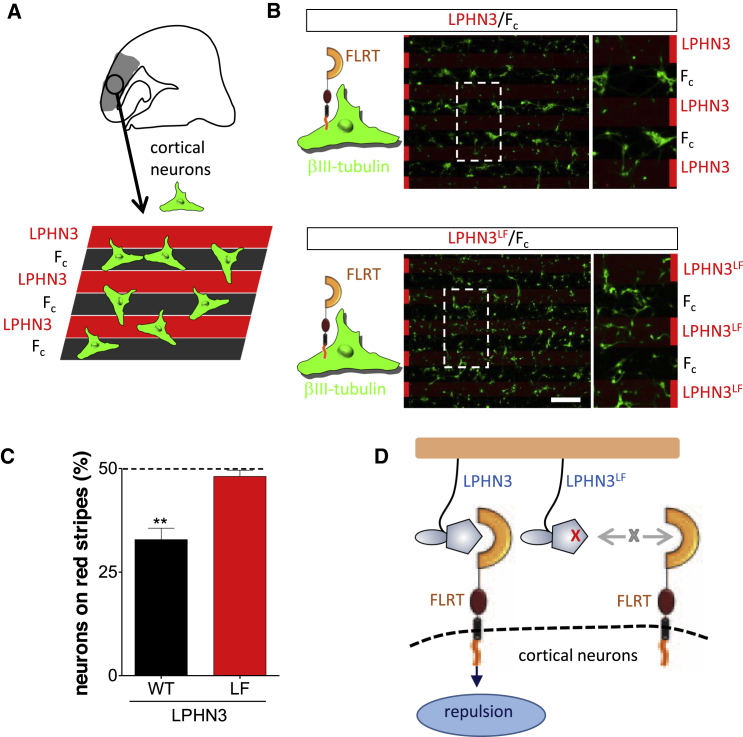
The FLRT-Binding Site of LPHN3^Lec-Olf^ Is Required for Repulsion of Cortical Neurons (A) Cartoon outlining the experimental setup for cortical neurons grown on alternating LPHN3 and F_c_ stripes. (B) Cortex-derived cultures (E15.5) were grown on alternating stripes of LPHN3^Lec-Olf^ and F_c_ control proteins. Cortical neurons expressing high levels of endogenous FLRTs (Seiradake et al., 2014) were immunostained for the neuron-specific βIII-tubulin (green).The neurons are repelled from LPHN3^Lec-Olf^, but not LPHN3^LF^ mutant, stripes. Insets are higher magnification images showing the distribution of cells on the stripes. Red bars indicate the locations of the red (LPHN3-containing) stripes. Scale bar, 200 μm. (C) Quantification of the data shown in (B), calculated for βIII-tubulin-stained neurons on LPHN3 stripes, essentially as done for GFP-expressing HeLa cells in Figure 4E. ^∗∗^p < 0.01, two-tailed Student t test. Error bars represent the SEM. (D) Cartoon summary depicting how wild-type LPHN3^Lec-Olf^, but not the FLRT-binding-impaired mutant, repels cortical neurons.

To assess the adhesive function previously postulated for the LPHN-FLRT interaction, we performed stripe assays in which HeLa cells transiently transfected with FLRT2-ires-GFP or the non-LPHN3-binding FLRT2 mutant (FLRT2^FF^-ires-GFP) chose between growth on alternating stripes of LPHN3^Lec-Olf^ and control protein (F_c_) (Figure 4A). For all experiments in Figure 4, LPHN3^Lec-Olf^-containing stripes were visualized by mixing the LPHN3^Lec-Olf^ wild-type or mutant proteins with Cy3-conjugated anti-hF_c_ antibody. The remainder of the dish was coated with control F_c_ protein and anti-hF_c_, resulting in the non-LPHN3^Lec-Olf^-coated areas appearing black. Expression of FLRT2 in HeLa cells led to a significant increase in the preference for growth on the LPHN3^Lec-Olf^ stripes (Figures 4B, 4E, and 4F). We also performed stripe assays in which FLRT2-transfected cells were asked to choose between growth on the non-FLRT-binding LPHN3 mutant protein, LPHN3^LF^, or control protein (Figures 4C, 4E, and 4F). In these experiments, the strong adhesive effect was abolished. Similarly, overexpression in HeLa cells of the non-LPHN3-binding FLRT2 mutant (FLRT2^FF^) led only to a mild preference for LPHN3^Lec-Olf^ stripes (Figures 4D and 4E). HeLa cells express endogenous FLRT1 and FLRT3 (data not shown), which may explain the small adhesive effect of LPHN3^Lec-Olf^ observed also in the presence of the mutant FLRT2. Thus our stripe assay data with transfected HeLa cells indicate that interaction between the LPHN3 Olf domain and the FLRTs promotes adhesion, consistent with the proposed role of LPHN3 as a positive regulator of synapse development (O’Sullivan et al., 2012). Next, we sought to test the behavior of cultured neurons expressing FLRTs on LPHN3^Lec-Olf^ stripes (Figure 5A). We chose cortical neurons, which express high levels of FLRT2 and FLRT3 (Seiradake et al., 2014). In contrast to HeLa cells, the neurons were repelled by LPHN3^Lec-Olf^ stripes (Figures 5B–5D). The repulsive effect was abrogated when the neurons were grown on non-FLRT-binding LPHN3^LF^ stripes (Figures 5B–5D), indicating that the FLRT-binding site on the LPHN3 Olf domain was responsible for the repulsive activity.

## Discussion

Our crystallographic data reveal the structure of a mammalian olfactomedin-like domain. The β-propeller fold reveals surprising structural homology with a range of enzymes, especially type I QCs from plant and bacteria. QCs catalyze the formation of pyroglutamate from glutamine or glutamate residues at the N terminus of proteins and peptides, and contain calcium or zinc in the catalytic site (Schilling et al., 2008a). Mammals express type II QCs, which are structurally unrelated and linked to neurodegenerative diseases such as Alzheimer’s disease (Schilling et al., 2008b). Given the structural similarity with the bacterial and plant enzymes, and the approximate conservation of the metal ions, it is tempting to speculate that the LPHN3 Olf also harbors an enzymatic activity. However, the ion-coordinating pockets are not structurally conserved between LPHN3 Olf and its closest structural relative, the QC from *X. campestris* (Figure S1C). Furthermore, a hydrophobic pocket located at the edge of the *X. campestris* QC active site, important for both substrate selection and the cyclization reaction (Huang et al., 2010), is markedly different in the LPHN3 Olf structure. In *X. campestris* QC, a glutamate residue (E89) acts as a general acid and base to assist in the intramolecular cyclization reaction. No such residue is found in the putative active site of LPHN3, meaning it is unlikely that LPHN3^Olf^ and bacterial QCs have exactly the same function. The “entrance” to the ion-binding pocket of LPHN3 Olf is obscured by loops. Should the LPHN3 Olf harbor an enzymatic function, the binding of substrate would likely involve structural rearrangement of these loops, perhaps providing substrate specificity. We expect that future studies will shed light on this exciting question.

Among other mammalian Olf-containing protein families, the ion-coordinating residues of LPHN are conserved in at least three: myocilin, olfactomedin-like 2, and, to a lesser degree, noelins (Figure 1D). Indeed, previous biophysical analysis suggested that myocilin binds calcium (Donegan et al., 2012). Mutation of residue D380, which is equivalent to LPHN3 D332, leads to loss of bound calcium (Donegan et al., 2012). Interestingly, the myocilin mutant D380A leads to the development of glaucoma (Donegan et al., 2012), which is characterized by the progressive degeneration of the optic nerve (Stone et al., 1997). Thus the calcium-binding site we have described here for LPHN is of critical functional relevance, at least in myocilin. Whereas in myocilin, olfactomedin-like 2, and LPHNs the metal-binding site appears fully conserved, noelins contain an aspartic acid in the place of mLPHN3 N380. As a result, the predicted ion-binding pocket in noelins is likely to be more acidic, although it could possibly still bind calcium. Olfactomedin 4 contains an asparagine in the place of LPHN3 D332 (or myocilin D380), thereby lacking the negative charge that appears to be essential for calcium binding at that position. Olfactomedin-like 1, olfactomedin-like 3, and gliomedins differ even more substantially at the putative ion-binding site, making it unlikely that they contain calcium or sodium at exactly the same positions as LPHN3.

Our stripe assay data with transfected HeLa cells indicate that interaction between the LPHN3 Olf domain and the FLRTs promotes adhesion, consistent with the proposed role of LPHN3 as a positive regulator of synapse development (O’Sullivan et al., 2012). Surprisingly, confrontation of primary cortical neurons with stripes containing LPHN3^Lec-Olf^ revealed that LPHN3 can be repulsive under certain conditions. This repulsive activity likely resides in the FLRT-binding site of the molecule, since the non-FLRT-binding mutant LPHN3^LF^ failed to be repulsive. How could FLRT proteins expressed on the growth cones of neurons mediate repulsion? Engagement of LPHN3 with FLRTs may recruit in *cis* and activate other guidance receptors with repulsive activity, such as Robo1 (Leyva-Díaz et al., 2014), Unc5 (Seiradake et al., 2014), or as yet unidentified receptors. Alternatively, LPHN3 binding to FLRTs may activate FLRT reverse repulsive signaling via its cytoplasmic tail, analogous to ephrin reverse signaling (Klein, 2012). Although the exact mechanism remains to be discovered, these findings clearly suggest that conserved surfaces on the LPHN3 Olf domain are critical for LPHN3 to act as a bi-functional protein with adhesive and repulsive properties.

## Experimental Procedures

### Cloning and Crystallography

Murine LPHN3 (LPHN3^Lec-Olf^ for crystallization (residues 92–463) and LPHN3^Olf^ (residues 202–463) were cloned into the pHLSec vector and transiently expressed in kifunensine-treated HEK293T cells using previously described methods (Aricescu et al., 2006) (see Supplemental Experimental Procedures for protein expression and crystallization details). X-Ray diffraction data were collected from native and derivative crystals at the peak wavelength of the platinum L-II edge at the Diamond Light Source beamline I04, processing the data using Xia2 (Winter et al., 2013). Autosharp (Vonrhein et al., 2007), Autobuster (Blanc et al., 2004), and Coot (Emsley and Cowtan, 2004) were used to solve the structure of LPHN3^Lec-Olf^ (see Supplemental Experimental Procedures).

For binding studies, the full-length FLRT2 ectodomain (residues 35–540) was cloned into the same vector and transiently expressed in HEK293S GlnTI^-^ cells.

### SPR

Equilibrium experiments were performed at 25°C using a Biacore T200 instrument (GE Healthcare). The experiments were carried out at pH 7.5 (PBS, 0.005% (v/v) polysorbate 20). The regeneration buffer was 2 M MgCl_2_. FLRT2 ectodomain proteins were coupled to CM5 Biacore chips via direct amine coupling. LPHN3^Lec-Olf^ proteins were biotinylated enzymatically at a C-terminal avidity tag (Avi-Tag) and attached via the resulting biotin label to streptavidin-coated Biacore chip surfaces to mimic the native membrane insertion topology. Data were analyzed with Scrubber2 (BioLogic). *K*_d_ and maximum analyte binding (*B*_max_) values were obtained by nonlinear curve fitting of a 1:1 Langmuir interaction model (bound = *B*_max_/(*K*_d_ + *C*), where *C* is analyte concentration calculated as monomer).

### Protein Cell Binding

HeLa cells in six-well plates were transfected with 2.0 μg of pCAGIG vector DNA encoding full-length FLRT2 using FuGENE 6 transfection reagent (Promega), according to the manufacturer’s instructions. Thirty-six hours after transfection, cells were incubated for 20 min at 4°C in Hank’s balanced salt solution containing LPHN3^Lec-Olf^ or LPHN3^Lec-Olf^ LF mutant protein pre-clustered (ratio 1:4) with His-probe rabbit polyclonal immunoglobulin G (IgG) (Santa Cruz Biotechnology). Cells were washed twice with PBS and fixed with 4% paraformaldehyde for 20 min at 4°C. Bound LPHN3^Lec-Olf^ proteins were then visualized with Alexa561-conjugated anti-rabbit IgG antibody (Molecular Probes, 1:400) after 30 min of blocking with 1% BSA. Nuclei were counterstained with DAPI before mounting.

### Stripe Assays

Stripe assays were prepared essentially as previously described (Yamagishi et al., 2011). In brief, stripe matrices were placed on 60-mm dishes and proteins were injected. After 30 min incubation at 37°C, dishes were washed with PBS and matrices were removed. The dishes were coated with F_c_ protein mixed with anti-hF_c_ for 30 min at 37°C, washed with PBS and, for culturing neurons only, coated with 20 μg/ml of laminin overnight at 37°C. HeLa cells or dissociated cortical (E15.5) neurons were cultured on the stripes for 3 hr (HeLa cells) or 16 hr (neurons), fixed, and stained. Details are provided in the Supplemental Experimental Procedures.

## Author Contributions

V.A.J. performed the crystallographic analysis, protein engineering, SPR experiments, and cell stripe assays. D.dT. aided and oversaw all stripe assay experiments. M.C. purified proteins for functional analysis. P.R. analyzed the LPHN3 ion-binding sites. K.H. performed heavy atom soaking and aided data collection and processing. E.S. produced protein crystals and oversaw biophysical experiments. All authors contributed to discussion and writing of the manuscript.

## Accession Numbers

The RCSB PDB accession number for the Latrophilin 3 structure reported in this paper is 5afb.

## Supplemental Information

Document S1. Figure S1, Table S1, and Supplemental Experimental Procedures

Document S2. 3D Molecular Model of LPHN3 Lec-Olf Domains

Document S3. Article plus Supplemental Information
