# Supplementary material for: Structural Basis of Latrophilin-FLRT Interaction
Source: Structure. 2015 Apr 7;23(4):774–81. doi: 10.1016/j.str.2015.01.013 (PMC4396693; doi:10.1016/j.str.2015.01.013)
Supplement: Document S1. Figure S1, Table S1, and Supplemental Experimental Procedures [file mmc1.pdf]

Structure, Volume *23*

Supplemental Information

Structural Basis of Latrophilin-FLRT Interaction

Verity A. Jackson, Daniel del Toro, Maria Carrasquero, Pietro Roversi, Karl Harlos, Rüdiger Klein, and Elena Seiradake

Supplemental Figure:

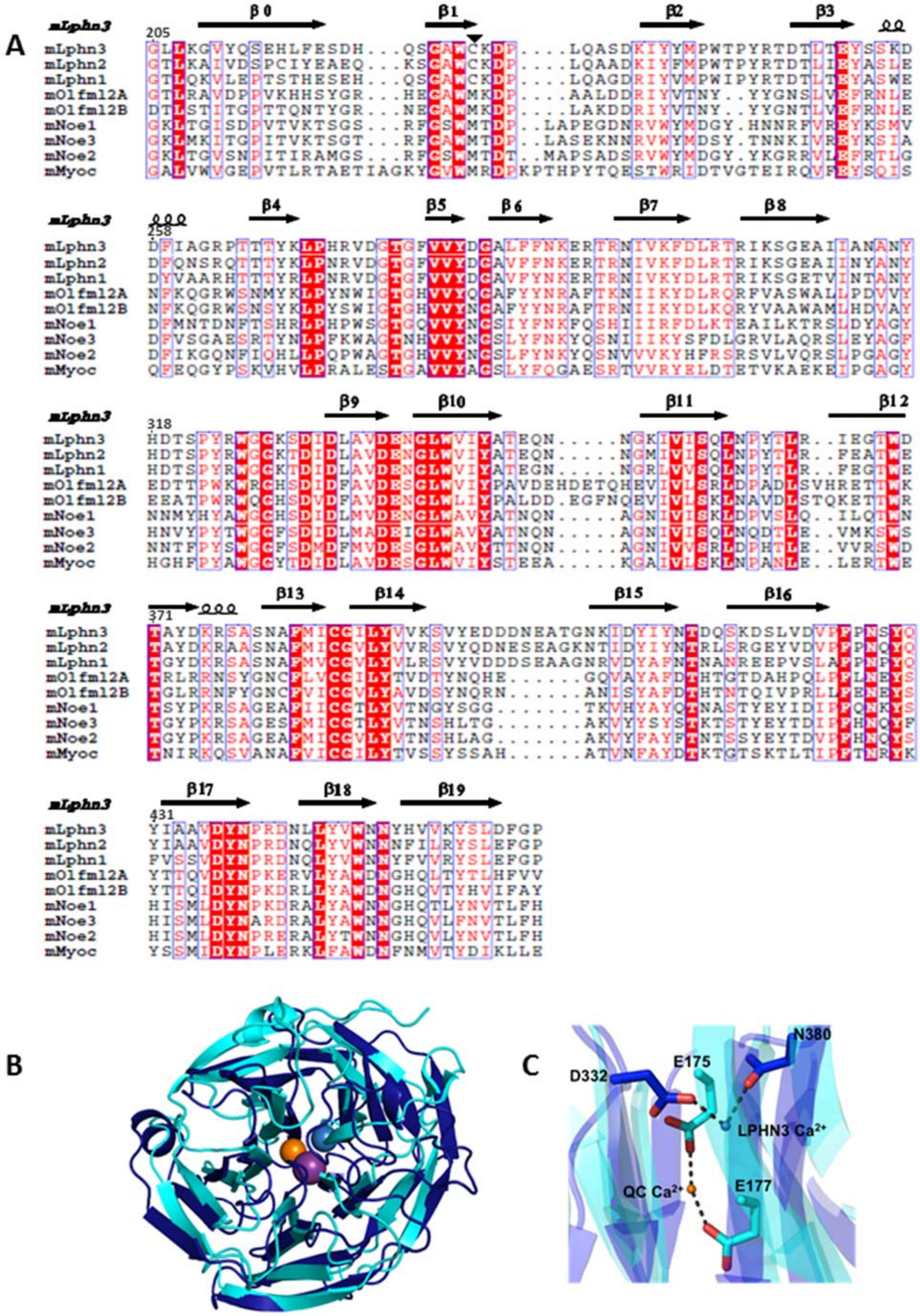

Figure S1, related to Figure 1. Sequence alignment of selected murine olfactomedin-like (Olf) domains and superposition of the mLPHN3 Olf and a glutaminyl cyclase from *Xanthomonas campestris*.

A Sequences from murine Latrophilins (mLPHN1-3), olfactomedin-like 2 (mOlfml2A,B), noelins (Noe1-3) and myocilin (mMyoc) were aligned and coloured according to sequence conservation using default parameters in ESPRIPT (Gouet et al., 2003). The unpaired cysteine 227 found in mLPHNs is marked with a black arrow head.

B LPHN3 Olf domain is shown in dark blue, with the sodium and calcium ions coloured in purple and skyblue, respectively. The structure of the *X.campestris* glutaminyl cyclase (PDB accession code 3MBR) (Huang et al., 2010) is shown in cyan, with the active site calcium ion shown in orange.

C Close-up view of the calcium-binding sites in the aligned LPHN3 Olf domain and *X.campestris* glutaminyl cyclase (QC) structures highlights the structural differences. The Ca^{2+}-coordinating residues E175 and E177 of *X.campestris* QC are sequence conserved among other bacterial and plant QCs, but not in LPHN3 Olf. Ca^{2+}-coordinating side chains are shown as sticks and aligned structures are shown in transparent cartoon representation. The colour scheme is as in panel B.

Supplemental Table:

	LPHN3$^{Lec-Olf}$ (native)	LPHN3$^{Lec-Olf}$ (K_2PtCl_6 derivative)
**Data collection**		
Wavelength (Å)	1.0711	1.0716
Space group	*I222*	*I222*
Cell dimensions		
a, *b*, *c* (Å)	78.4, 96.6, 101.64	77.09, 97.15, 101.66
α, β, γ (°)	90, 90, 90	90, 90, 90
Crystal solvent content (%)	46	46
Resolution (Å)*	62-2.16 (2.22-2.16)	70-2.6 (2.66-2.6)
I / σ*I**	10.2 (1.7)	14.7 (1.7)
Completeness (%)*	99.5 (98.9)	99.5 (95.0)
Redundancy*	3.49 (3.5)	32.17 (18.36)
R-meas (%)	10.4 (86.1)	25.3 (174.9)
**Refinement**		
Resolution (Å)*	62.1-2.16 (2.27-2.16)	-
No. of reflections*	21031 (2605)	-
R_{work} / $R_{free}^{\#}$	0.205 / 0.244	-
**No. of atoms** -		
Protein	2814	-
Heterogen	116	-
Water	88	-
Average *B*-factor	42.7	-
R.m.s. deviations		
Bond lengths (Å)	0.023	-
Bond angles (°)	1.47	-
Ramachandran plot for residues		
Favoured regions (%)	95.01	-
Allowed regions (%)	4.99	-
Disallowed regions (%)	-	-

Values in parentheses are for the highest-resolution shell.

#For the calculation of *Rfree*, 5% of reflections across all resolution shells were randomly assigned and used only for validation. *Rfree* = ΣTest||*F*obs| −|*F*calc||/ΣTest |*F*obs|.

Table S1, related to Figure 1. Crystallographic statistics.

Supplemental Experimental Procedures.

Protein purification and crystallisation. Cell culture medium containing recombinant proteins was collected and cleared by centrifugation and filtration. Secreted recombinant proteins were isolated from conditioned medium by immobilized metal affinity chromatography and purified further by size-exclusion chromatography in 10 mM Tris-HCl (pH 8.0), 150 mM NaCl. Prior to crystallization, recombinant endoglycosidase F1 (Chang et al., 2007) was added at a concentration of 0.01 mg/ml to all samples. $LPHN3^{Lec\text{-}Olf}$ was concentrated to 9.3 mg/ml in 150 mM NaCl, 10 mM Tris-HCl pH 8.0. Crystals were grown by the vapour diffusion method at 20 °C by mixing protein and crystallisation solution (0.1 M MES buffer pH 6, 4M NaCl, 0.6 M non-detergent sulfo-betaine NDSB195) in a 1:1 (v/v) ratio. The crystals were flash-frozen in a cryoprotectant solution containing 75% crystallisation solution and 25% glycerol. To produce Pt derivative crystals, K_2PtCl_6 was added in powder form to the drop containing $LPHN3^{Lec\text{-}Olf}$ crystals and the crystals were frozen in cryoprotectant solution six hours later.

X-ray diffraction data processing. One native and one Pt-derivative dataset were collected from $LPHN3^{Lec\text{-}Olf}$ crystals and used as input for single isomorphous replacement phasing in Autosharp (Vonrhein et al., 2007). Autosharp identified five platinum positions and performed automatic phasing and model building. The model was refined against the native data with Autobuster (Blanc et al., 2004) and further manually built in Coot (Emsley and Cowtan, 2004). Programs from the Collaborative Computational Project 4 (CCP4) and Molprobity (Davis et al.,

2007) were used to validate the resulting structure. Crystallographic details are summarized in Table 1. Root mean square deviations (rmsd) between C-alpha atoms were calculated with Superpose (Maiti et al., 2004). The identities of the metal-ion sites were inferred using calcium bond-valence sum (CBVS), a convenient method for analysis of the geometric environment of potential sites with a view to metal-ion assignment (Müller et al., 2003). Analysis of the ion-coordination geometries in the LPHN3$^{Lec-Olf}$ crystal was performed by implementing formula (3) of reference (Müller et al., 2003) in a simple Unix shell script, and computing the value of CBVS from the experimentally observed ligand occupancies and ligand-ion bond distances. These values were used to infer the ions' chemical identities by comparison with the expected CBVS values ($CBVS_{Ca++}$=2.0 and $CBVS_{Na+}$=1.57).

Stripe assays. 50 µg/ml of LPHN3$^{Lec-Olf}$ (wild type or mutant) were mixed with Cy3-conjugated anti-hF_c antibody (Invitrogen) in PBS. Matrices (90 µm width) were placed on 60 mm dishes (Knöll et al., 2007) and proteins injected, resulting in red fluorescent stripes. After 30 min incubation at 37°C, dishes were washed with PBS and matrices were removed. The dishes were coated with 50 µg/ml of F_c protein mixed with anti-hF_c for 30 min at 37°C and then washed three times with PBS and, for culturing neurons only, coated with 20 µg/ml of laminin overnight at 37°C. HeLa cells (30,000 cells/experiment) or dissociated cortical (E15.5) neurons (50,000 cells/experiment) were cultured for 3 h (HeLa cells) or 16 h (neurons) on the stripes. Samples were fixed with 2% sucrose/4% PFA in PBS for 10-20 min at room temperature (RT). Cells were washed and the nuclei counterstained with DAPI before mounting. Neurons were washed and incubated with mouse monoclonal anti- beta-III tubulin antibody (Sigma) after 10 min permeabilization in 1% BSA, 0.1% Triton X-100/PBS. Cy2 or Cy3 anti-rabbit IgG secondary antibody (Jackson) was used to visualize the tubulin signal. The number of GFP+ pixels on red stripes were quantified with ImageJ

References.

Blanc, E., Roversi, P., Vonrhein, C., Flensburg, C., Lea, S.M., and Bricogne, G. (2004). Refinement of severely incomplete structures with maximum likelihood in BUSTER-TNT. Acta Crystallogr. D. Biol. Crystallogr. *60*, 2210–2221.

Chang, V.T., Crispin, M., Aricescu, A.R., Harvey, D.J., Nettleship, J.E., Fennelly, J.A., Yu, C., Boles, K.S., Evans, E.J., Stuart, D.I., et al. (2007). Glycoprotein structural genomics: solving the glycosylation problem. Structure *15*, 267–273.

Davis, I.W., Leaver-Fay, A., Chen, V.B., Block, J.N., Kapral, G.J., Wang, X., Murray, L.W., Arendall, W.B., Snoeyink, J., Richardson, J.S., et al. (2007). MolProbity: all-atom contacts and structure validation for proteins and nucleic acids. Nucleic Acids Res. *35*, W375–W383.

Emsley, P., and Cowtan, K. (2004). Coot: model-building tools for molecular graphics. Acta Crystallogr. D. Biol. Crystallogr. *60*, 2126–2132.

Gouet, P., Robert, X., and Courcelle, E. (2003). ESPript/ENDscript: Extracting and rendering sequence and 3D information from atomic structures of proteins. Nucleic Acids Res. *31*, 3320–3323.

Huang, W.-L., Wang, Y.-R., Ko, T.-P., Chia, C.-Y., Huang, K.-F., and Wang, A.H.-J. (2010). Crystal structure and functional analysis of the glutaminyl cyclase from Xanthomonas campestris. J. Mol. Biol. *401*, 374–388.

Knöll, B., Weinl, C., Nordheim, A., and Bonhoeffer, F. (2007). Stripe assay to examine axonal guidance and cell migration. Nat. Protoc. *2*, 1216–1224.

Maiti, R., Van Domselaar, G.H., Zhang, H., and Wishart, D.S. (2004). SuperPose: a simple server for sophisticated structural superposition. Nucleic Acids Res. *32*, W590–W594.

Müller, P., Köpke, S., and Sheldrick, G.M. (2003). Is the bond-valence method able to identify metal atoms in protein structures? Acta Crystallogr. D. Biol. Crystallogr. *59*, 32–37.

Vonrhein, C., Blanc, E., Roversi, P., and Bricogne, G. (2007). Automated structure solution with autoSHARP. Methods Mol. Biol. *364*, 215–230.
